# Development of *Leishmania mexicana* in *Lutzomyia longipalpis* in the absence of sugar feeding

**DOI:** 10.1590/0074-02760180482

**Published:** 2019-05-20

**Authors:** Samara G da Costa, Caroline da Silva Moraes, Paul Bates, Rod Dillon, Fernando Ariel Genta

**Affiliations:** 1 Fundação Oswaldo Cruz-Fiocruz, Instituto Oswaldo Cruz, Laboratório de Bioquímica e Fisiologia de Insetos, Rio de Janeiro, RJ, Brasil; 2 Lancaster University, Faculty of Health and Medicine, Division of Biomedical and Life Sciences, Lancaster, United Kingdom; 3 Instituto Nacional de Ciência e Tecnologia em Entomologia Molecular, Rio de Janeiro, RJ, Brasil

**Keywords:** Leishmania mexicana, Lutzomyia longipalpis, sugar

## Abstract

The leishmaniases are caused by *Leishmania* parasites and transmitted through the bites of phlebotomine sand flies. During parasite development inside the vector’s midgut, promastigotes move towards the stomodeal valve, a mechanism that is crucial for transmission. It has been reported that the sugar meal acquired by sand flies during feeding between bloodmeals is essential for the development and migration of parasites. We demonstrated that the distribution of *Leishmania mexicana* parasites was affected by the sugar meals obtained by the sand flies. Promastigote migration towards the cardia region seems to be only partially based on the stimuli provided by sugar molecules. In the absence of sugars, significant amounts of parasites developed in the hindgut. In addition, sugar meals were important for the survival of sand flies, especially during blood digestion, presumably supporting their energy requirements.

*Leishmania* parasites develop as extracellular forms (promastigotes) in the gut of their sand fly vectors and as obligate intracellular forms (amastigotes) inside the phagolysosomes of infected macrophages in the vertebrate host. The development of *Leishmania* parasites inside the vector is complex and dynamic. Depending on the *Leishmania* subgenus a different pattern of development can be observed inside the gut of the vector, for *Leishmania mexicana* (subgenus *Leishmania*) parasites develop exclusively in the midgut and foregut of their vectors, which is known as suprapylarian development.^1^

After ingestion of an infective blood meal by the sand fly, macrophages containing parasites release their amastigotes forms into the blood meal, and the change in pH conditions triggers the differentiation of amastigotes into promastigotes,^2^ a motile and replicative form. These parasites have different developmental stages inside the gut of the vector. For infection establishment, two cycles of multiplication occur during parasites development. The first cycle occurs with the multiplication of procyclic promastigotes inside the peritrophic matrix, in the blood meal phase.^3^ ^)^ After the digestion of blood, parasites escape from the peritrophic matrix, attach to the midgut epithelium and migrate to the anterior midgut region.^4^ ^)^ The second cycle of multiplication takes place in the sugar meal phase with the leptomonad promastigotes, which differentiate in the non-multiplicative infective metacyclic promastigote forms. It is hypothesised that the presence of sugar ingested by the female sand fly between bloodmeals triggers the multiplication of leptomonad promastigotes.^3^

Between blood meal feeds, sand flies take sugar-rich meals that are stored in the crop.^5^ ^)^ The sugar meal is then released in small quantities into the midgut. After blood meal digestion, the sugar meal rich that can contain sucrose, raffinose, melezitose, starch, and cellulose (besides other types of sugars) is a potential source of nutrition for parasites developing inside the vector gut. It is believed that the ingestion of sugar by the vector impacts the developing promastigote parasite population. ^6^ ^,^ ^7^ ^,^ ^8^ ^)^ It was described for different *Leishmania* species that they secrete glycosidases, enzymes specialised in the digestion of sugars, like alpha-glucosidase, sucrases, invertases, alpha-amylases, and others.^8^ ^,^ ^9^ ^,^ ^10^ ^,^ ^11^ ^)^ For *L. mexicana*, both invertase and sucrase activity were identified as secreted by promastigotes.^7^ ^,^ ^10^ ^)^ In this respect, *L. mexicana* might use sugar meals as an exogenous source of energy for its development.

In addition, sugar ingestion by females sand flies creates a sugar gradient along the midgut, and it was reported that this gradient provides the stimulus for parasite migration towards the stomodeal valve region (critical for efficient transmission) by mechanisms of chemo- and osmotaxis.^12^ ^,^ ^13^ ^,^ ^14^ ^,^ ^15^ However, studies investigating the effects of the sugar meal on parasite migration and development using an in vivo model need to be performed.

In this work, we demonstrated that the distribution of *Leishmania mexicana* along the gut of *Lutzomyia longipalpis* is reliant on sugar feeding by phlebotomines. In the absence of sugar meals, although the parasites are capable of reaching the stomodeal valve region, a significant population of parasites instead develop in the hindgut of the insect. Also, although sugar feeding was not necessary for the complete development of parasites, the survival of *Lu. longipalpis* was drastically affected by the absence of sugar feeding, especially after blood-feeding. In this respect, we emphasise the importance of sugar meals during the life cycle of both sand fly vectors and *Leishmania* parasites.

For this investigation insectary-reared *Lu. longipalpis* (Jacobina, Bahia, Brazil), maintained at Lancaster University (United Kingdom), were used for experiments. Insects were kept under standard laboratory as described in Moraes et al.^16^ For experiments, groups of recently emerged females (0 - 3 hours) were separated into small cages, kept for three days with access to water only, followed by blood feeding or infected blood feeding using a Hemotek apparatus (Discovery Workshops), with chicken skin membranes held at 37ºC for 1 hour, and then maintained under different conditions with access to water or 1.2 M sucrose.

In this study, *L. mexicana* (World Health Organization strain MNYC/BZ/1962/M379) from an axenic culture of amastigote-like forms was used for infections. Amastigote-like culture and sand fly infections were performed as described by Moraes et al.^16^ ^)^ For infections, a concentration of 2 x 10^6^ parasites/mL, estimated with Neubauer chambers, was used. Briefly, after centrifugation at 2000 x g for 5 min, the supernatant was removed, and parasites were mixed with sheep blood and offered to 3-day old females maintained with water (unfed). After blood feeding, unfed females were discarded, and the fed ones were kept with water only or 1.2 M sucrose.

For estimation of *L. mexicana* infections, the whole gut of infected females was dissected and analysed under light microscopy at three, six and 10 days after blood feeding to check for establishment. Dissections were conducted in PBS on microscope slides using needles. Dissected guts were transferred to polypropylene tubes containing 20 µL PBS and 2% paraformaldehyde, used to immobilise parasites. After homogenisation and dilution, a 10 µL sample was transferred to Neubauer chambers, and the total number of parasites was determined. We also analysed the number of metacyclic promastigotes in day six samples. The identification of metacyclic promastigotes followed the characteristics described for the identification of *Leishmania* different developmental stages.^17^ On the third and sixth days after the blood feeding, the number of parasites was also estimated in the hindgut and midgut, separately, using the same procedure described above.

We also analysed the longevity of *Lu. longipalpis* under different conditions. Mortality was evaluated, and dead insects were removed from cages daily. For each biological replicate and condition tested 100 females were used. As specified above, emerged females (0-3 hours) were separated, and six different feeding conditions were monitored. The following groups were analysed: unfed maintained with water, fed on 1.2 M sucrose (SF), blood-fed (infective meal or not) maintained with 1.2 M sucrose or blood-fed (infective meal or not) maintained with water.

All statistical analysis of parasite infections was performed with GraphPad Prism 6.0 for Windows (San Diego, California, USA), and the D’Agostino-Pearson Omnibus K2 normality test was used. The outliers were identified with the ROUT method, and Q was established as 1%. One-way ANOVA (multiple comparisons) followed by Tukey’s multiple comparison tests and significance was considered when p < 0.05. For survival, results were analysed using the Kaplan-Meier survival curve obtained with GraphPad Prism 6.0 for Windows (San Diego, California, USA) and thus the average survival time was determined in each condition. The log-rank Mantel-Cox test was used to compare survival curves. Significance was considered when p < 0.05.

Our results demonstrated that the number of parasites present in the whole gut was not affected by the presence of sucrose. Comparisons were performed three and six days after infection (Fig. 1). Due to high mortality, it was not possible to evaluate infections at 10 days in water-fed females. Furthermore, the number of parasites did not increase following the days after infection for either water or sucrose fed females. After six days, we also analysed the number of *Leishmania* metacyclic forms in the midgut (including stomodeal valve), and no significant difference was detected in the numbers when comparing water fed to 1.2 M sucrose fed females, with 1200 ± 200 and 1400 ± 200 metacyclic promastigotes per midgut, respectively. So, we demonstrate that *L. mexicana* can develop inside *Lu. longipalpis*, even in the absence of sugar feeding by the phlebotomine host. There was no significant difference comparing the total number of parasites inside the gut, or the number of metacyclic forms, in water or sugar-fed females. Previous works discuss the importance of sugar feeding by phlebotomine sand flies for parasite development.^6^ ^,^ ^7^ ^,^ ^8^ ^,^ ^10^ ^)^ According to them, *Leishmania* promastigotes depend on the diet of their phlebotomine host to sustain their growth. Sugars may also prevent the egestion of *Leishmania* during defecation of blood meal remnants.^18^

**Fig. 1:**
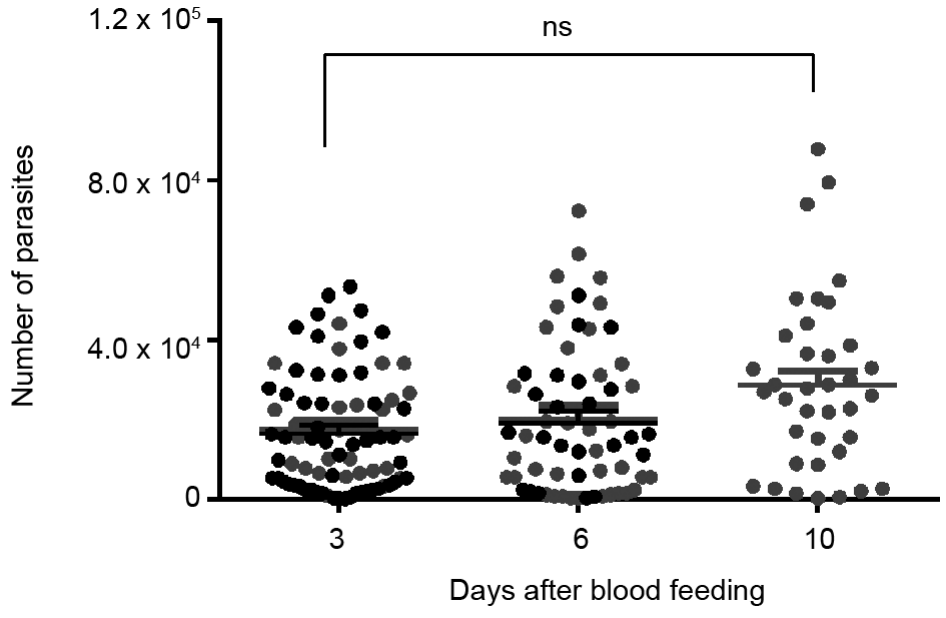
*Leishmania mexicana* parasites quantification in the gut of *Lutzomyia longipalpis* females at different days following the blood feeding. Recently emerged (0-3 h) females were collected and maintained with water for three days before feeding with an infected blood meal. After feeding, insects were maintained with sucrose 1.2 M (grey circles) or water (black circles). Circles represent the number of parasites per individual gut. The results are the mean ± SEM of two independent experiments. One-way analysis of variance (ANOVA) was performed followed by Tukey multiple comparison test. ns: non-significant difference.

In our work, we demonstrated the multiplication and development of promastigote and metacyclic forms in the guts of water-fed flies, although the infectivity of these parasites in a second blood meal remains to be addressed. In addition, we do not know if the absence of sugar can affect the appearance of different promastigotes forms during development. The development of *L. mexicana* parasites into different promastigotes forms was described as sugar dependent.^3^ ^)^ Protocols for studying the effect of a second blood meal in *Lu. longipalpis* under laboratory conditions have been recently developed^16^ and it has been recently demonstrated that the ingestion of a second non-infecting blood meal by infected sand flies leads to enhanced disease transmission by amplifying the number of parasites acquired in the infected blood meal. The process occurs by dedifferentiation of the metacyclic promastigotes into replicative “retroleptomonad” promastigote forms, which leads to increased infection.^19^ ^)^ Although components present in plasma were reported to trigger dedifferentiation, we cannot rule out the hypothesis that sugar absence might also have an effect in the appearance of promastigote retroleptomonad forms or affect the numbers of parasites in the case of a second blood meal. So, we expect to extend these observations in the future.

Our data suggest that nutrients obtained from sugar meals are not strictly necessary for parasite growth and differentiation, and the parasites are presumably obtaining nutrients released from blood hydrolysis in the absence of a sugar meal. The nutrients obtained from blood are likely to be especially necessary for the early phases of development when the parasite is trapped inside the peritrophic matrix. Differently from the results demonstrated in our work, in infections of *Lu. longipalpis* with *Leishmania donovani*, a regular sugar meal was shown to enhance the number of parasites inside the gut of the vector,^20^ ^)^ and also in *Lu. youngi* the efficiency of infection with *L. amazonensis* was affected by the type of sugar used to feed the sand flies.^6^ ^)^ It is possible that, under our conditions, the presence or absence of sugar meals impacts the development and survival of parasites only after more extended periods after the blood feed, and further studies must confirm or reject this hypothesis.

In our work, although no differences were found in the total number of parasites after different feeding regimes, we observed a difference in the pattern of parasite distribution. In females fed with water after three and six days of infection, we found a large number of parasites in the hindgut. In Fig. 2, we present images obtained by light microscopy demonstrating the presence of parasites in the hindgut of water-fed females (Fig. 2A-B), with many parasites in this region. The hindgut of these insects is filled with parasites, but these do not seem to be attached to the cuticle (Supplementary data). In water-fed females, parasites were also present in the midgut and cardia region (Fig. 2C), while for sugar-fed females parasites are not distributed along the hindgut (Fig. 2D). Considering the migration of parasites to the hindgut, we evaluated the number of parasites in this compartment in water and sucrose-fed females. The percentage of infected females presenting parasites in the hindgut was larger for water-maintained flies. After three days post-blood feeding 70% of analysed insects had parasites in the hindgut compared with 20% in sugar-maintained insects (Fig. 3A). Six days after blood-feeding almost 90% of water-maintained females had parasites in the hindgut (Fig. 3A). For the midgut, the number of parasites (Fig. 3B) was consistent with the same pattern demonstrated in Fig. 1, in both water and sugar-maintained insects a massive number of parasites concentrated in this region. In contrast, although the absolute numbers recorded are lower, the number of parasites in the hindgut of water-fed females was significantly higher compared to sucrose-fed females (Fig. 3C), even though, the parasites number in the hindgut did not increase from three to six days. However, the data obtained in these assays suggest that the number of parasites quantified in the hindgut of water-fed flies, compared to what we can observe in the images (Fig. 2B), was underestimated, possibly due to the limitation of the technique of rupturing the gut for separation of midgut and hindgut.

**Fig. 2:**
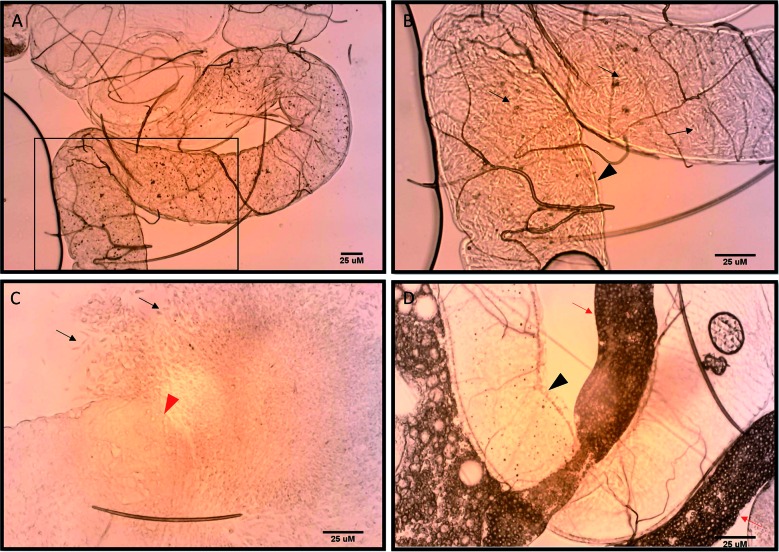
light microscopy images of infected *Lutzomyia longipalpis* females six days after blood feeding. Recently emerged (0-3 h) females were collected and maintained with water for three days before feeding with an infected blood meal. After feeding, insects were maintained with water (A, B, C) or sucrose 1.2 M (D). (A) hindgut 20 X magnification of infected water-maintained females. (B) 40 X magnification of delineated section from Fig. 2A. (C) 40 X magnification of the cardia of infected water-maintained females. (D) hindgut 40 X magnification of infected sugar-maintained females. Note gut epithelium (black arrowhead), *Leishmania* parasites (black arrows), Malpighian tubules (red arrows) and cardia (red arrowhead).

**Fig. 3:**
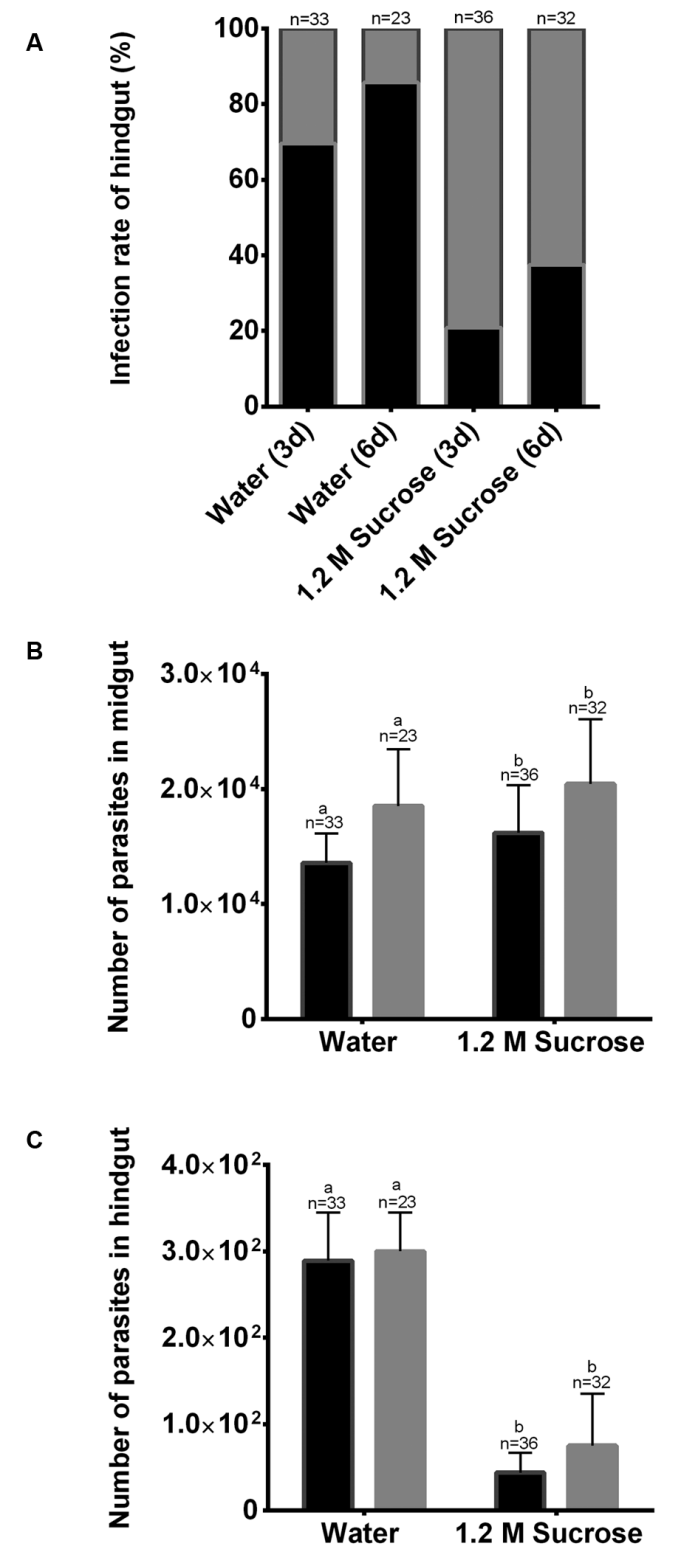
*Leishmania mexicana* parasites quantification in the midgut and hindgut of *Lutzomyia longipalpis* after three and six days following blood feeding. Recently emerged (0-3 h) females were collected and maintained with water for three days before feeding with an infected blood meal. After feeding, insects were maintained with sucrose 1.2 M or water. (A) Infection rate of hindgut of water or sugar-maintained females. The black background indicates the percentage of positive samples containing parasites in the hindgut and the grey background represents the percentage of negative samples. (B) Quantification of parasites in the midgut of water or sugar-maintained females after three days (black bars) and six days (grey bars). (C) Quantification of parasites in the hindgut of water or sugar-maintained females after three days (black bars) and six days (grey bars). One-way analysis of variance (ANOVA) was performed, followed by Tukey multiple comparison tests. Different letters indicate statistically significant differences in quantification, p < 0.001.

**Fig. 4:**
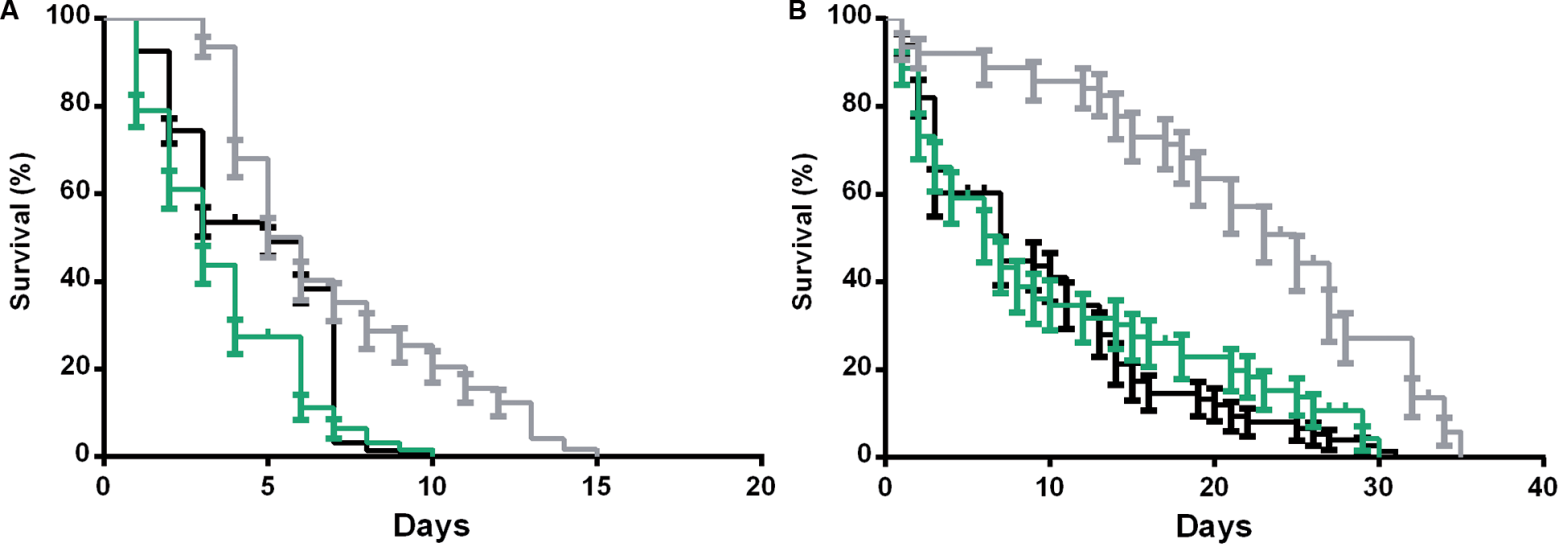
survival curve of *Lutzomyia longipalpis* females in different feeding conditions, maintained under controlled humidity and temperature conditions. Recently emerged (0-3 h) females were collected and maintained with water for three days before feeding with an uninfected or infected blood meal. After blood feeding (infected or not), insects were maintained with sucrose 1.2 M or water. Control groups were only fed with 1.2 M sucrose or water (no blood meal). (A) Non-fed females maintained with water (grey line), blood-fed females maintained with water post blood feeding (green line), infective blood-fed females maintained with water after infection (black line). (B) 1.2 M sucrose fed females (grey line), blood-fed females maintained with 1.2 M sucrose post blood feeding (green line), infective blood-fed females maintained with sucrose 1.2 M after infection (black line). The results are representative of three independent experiments. For each replicate, at least, 100 females were used. Log-rank Mantel-Cox test was performed, and the survival curves were significantly different at p < 0.0001.

During the development of parasites inside the gut of the vector, the movement of promastigotes to the anterior region of the sand fly midgut, with the accumulation of metacyclic promastigotes in the stomodeal valve, is critical, causing a distension of the valve and transmission to a mammal when a next blood-feeding occurs.^2^ ^,^ ^17^ Taxis is a phenomenon where an organism responds to specific stimuli by movement. It was proposed that during development the promastigotes could be attracted by the sugar meals ingested by sand flies, then migrating to the anterior region of the midgut.^21^ ^)^ Some works described that *Leishmania* promastigotes undergo chemotaxis in a gradient constituted of different sugars^13^ ^,^ ^15^ and likewise by serum albumin, hemoglobin, besides others.^15^ ^)^ The movement is also due to the osmotic gradient generated by the presence of sugars.^12^ ^)^ For *L. amazonensis* it was demonstrated that the parasite was able to respond both to chemotactic and osmotactic stimuli.^14^ ^)^ In this respect, both mechanisms of chemotaxis and osmotaxis play a role in the direction of parasites to the stomodeal valve region. We demonstrated that parasites are more frequently found in the hindgut (not attached) of water-fed compared to sugar-fed females. However, in both conditions, a high number of parasites were also able to reach the stomodeal valve. In this respect, the presence of sugar, creating an osmotic and chemical gradient, seems to be important but not obligatory to direct the migration of parasites toward the cardia region. In normal conditions where the sugar concentration is much higher than the other components, it might function as the central stimulator for parasite migration. However, in a situation where a large quantity of sugars is not present, the movement towards the stomodeal valve might be explained by water flow or by the presence of other components inside the vector gut that might also create an orientation stimulus for parasite migration. The midgut of the vector is divided into specialised regions with a variety of chemical and structural features that *Leishmania* parasites might exploit for orientation. Some studies have demonstrated that chemotaxis in *Leishmania* could be elicited by a wide range of compounds,^13^ ^,^ ^15^ ^)^ and saliva components might also work as taxic agents. It was proposed that the receptors involved in chemotaxis possess low specificity and a wide range of affinity, the same receptor might be able to bind structurally related molecules.^14^

*Leishmania* parasites have been classified as suprapylarian, peripylarian or hypopylarian, based on the region of their development along the gut of the sand fly vector.^1^ ^)^ *Leishmania* species that exclusively develop in the gut regions anterior to the pylorus are considered suprapylarian and belong to the subgenus *Leishmania*. *Leishmania* species that also colonise the abdominal gut regions, around the pylorus, are named peripylarian, and belong to the New World *Viannia* subgenus. *Leishmania* species that develop mainly in the hindgut are named hypopylarian, and belong to the subgenus *Sauroleishmania* and infect reptiles. Interestingly, our data suggest that the distribution of *Leishmania* parasites along the gut of sand flies also depends on the sugar meal of the vector, as in our conditions, *L. mexicana*, a suprapylarian parasite from the subgenus *Leishmania*, shows considerable development in the hindgut in the absence of sugars in the phlebotomine diet.

Finally, we examined the longevity of *Lu. longipalpis* under different feeding conditions, and this demonstrated that the median survival was drastically reduced from 25 days to five days, for sucrose fed females compared to water fed (starving) insects (Fig. 4A-B). The results also demonstrate that blood-feeding detrimentally affects the survival of the sand flies, but not the presence of *L. mexicana* parasite, at least under these conditions. In sugar-fed females, the mean survival was reduced from 25 to seven days, almost a 70% reduction, in blood-fed females (infected or not infected) compared to the non-blood fed ones. For water-maintained females, the median survival was reduced from five to three days, in blood-fed females (infected or not infected), compared to the non-blood fed females.

Although the parasite does not seem to have an absolute requirement for sugar to undergo development in our conditions, sugar is essential for phlebotomine survival. Without sugar meals, the mortality of sand flies was drastically enhanced, especially when females were also blood-fed. Sugar feeding appears to be vital to the metabolic demands of phlebotomine sand flies. The glucose, for example, obtained from sugar hydrolysis could be taken up by enterocytes, and converted to trehalose or stored as glycogen to supply the energetic demands of insects, like flight. In a starving condition, the reserves of glycogen and triglycerides are mobilised.^22^ ^,^ ^23^ ^)^ During blood digestion, nutrients as heme and amino acids are present in excess, and these molecules need to be detoxified by disposal or converted to advantageous derivatives. The release of heme is toxic because it potentiates oxygen-reactive species and can permeate membranes.^24^ Moreover, there is enhanced microbial growth after blood feeding that needs to be controlled.^25^ ^)^ Briefly, we suggest that during the blood digestion, there is an energetic demand to maintain the homeostasis in the organism. In a starving phlebotomine sand fly, weakened by the lack of energy, the hazardous effects of molecules or pathogens increased during blood digestion would be enhanced, and the pathways used for detoxification of these compounds or control of pathogens might be restricted.

In this respect, according to the results reported here, the development and migration of *L. mexicana* towards the stomodeal valve region, a mechanism essential for transmission, is not strictly dependent on sugar feeding by the phlebotomine host, but the sugar meals are necessary to supply the energy requirements for the survival of sand flies, especially during blood digestion. The survival of sand flies for an extended time is crucial for *Leishmania* transmission to the mammalian host since a minimum of two blood feeds are necessary for this. Thus, even with the viable development of the parasites in the absence of sugar, the transmission cycle might not occur, because the sand flies do not survive long enough to perform two blood feeds.
